# Safety and tolerability of atogepant for the preventive treatment of migraine: a post hoc analysis of pooled data from four clinical trials

**DOI:** 10.1186/s10194-024-01736-z

**Published:** 2024-03-11

**Authors:** Paul Rizzoli, Michael J. Marmura, Jennifer Robblee, Jennifer McVige, Sara Sacco, Stephanie J. Nahas, Jessica Ailani, Rosa De Abreu Ferreira, Julia Ma, Jonathan H. Smith, Brett Dabruzzo, Messoud Ashina

**Affiliations:** 1 Brigham and Women’s Hospital, Boston, MA USA; 2 Department of Neurology, Thomas Jefferson University, Jefferson Headache Center, Philadelphia, PA USA; 3 Barrow Neurological Institute, Phoenix, AZ USA; 4 DENT Neurologic Institute, Amherst, NY USA; 5 Carolinas Headache Clinic, Matthews, NC USA; 6 MedStar Georgetown University Hospital, Washington, DC USA; 7 AbbVie, 1 N. Waukegan Rd, North Chicago, IL 60064 USA; 8 AbbVie, Florham Park, NJ USA; 9 Department of Neurology, Danish Headache Center, Copenhagen University Hospital – Rigshospitalet, Copenhagen, Denmark; 10 Department of Clinical Medicine, University of Copenhagen, Copenhagen, Denmark

**Keywords:** Calcitonin gene–related peptide, Migraine, Safety, Tolerability

## Abstract

**Background:**

Conventional, non-specific preventive migraine treatments often demonstrate low rates of treatment persistence due to poor efficacy or tolerability. Effective, well-tolerated preventive treatments are needed to reduce migraine symptoms, improve function, and enhance quality of life. Atogepant is a migraine-specific oral calcitonin gene–related peptide receptor antagonist that is indicated for the preventive treatment of migraine in adults. This analysis evaluated the safety and tolerability profile of atogepant for the preventive treatment of migraine, including adverse events (AEs) of interest, such as constipation, nausea, hepatic safety, weight changes, and cardiac disorders.

**Methods:**

This post hoc analysis was performed using data pooled from 2 (12-week) randomized, double-blind, placebo-controlled trials (RCTs) and 2 (40- and 52-week) open-label long-term safety (LTS) trials of oral atogepant for episodic migraine (EM).

**Results:**

The safety population included 1550 participants from the pooled RCTs (atogepant, *n* = 1142; placebo, *n* = 408) and 1424 participants from the pooled LTS trials (atogepant, *n* = 1228; standard care [SC], *n* = 196). In total, 643/1142 (56.3%) atogepant participants and 218/408 (53.4%) placebo participants experienced ≥ 1 treatment-emergent AEs (TEAEs) in the RCTs. In the LTS trials, 792/1228 (64.5%) of atogepant participants and 154/196 (78.6%) of SC participants experienced ≥ 1 TEAEs. The most commonly reported TEAEs (≥ 5%) in participants who received atogepant once daily were upper respiratory tract infection (5.3% in RCTs, 7.7% in LTS trials), constipation (6.1% in RCTs, 5.0% in LTS trials), nausea (6.6% in RCTs, 4.6% in LTS trials), and urinary tract infection (3.4% in RCTs, 5.2% in LTS trials). Additionally, weight loss appeared to be dose- and duration-dependent. Most TEAEs were considered unrelated to study drug and few led to discontinuation.

**Conclusions:**

Overall, atogepant is safe and well tolerated in pooled RCTs and LTS trials for the preventive treatment of EM in adults.

**Trial registration:**

ClinicalTrials.gov identifiers: NCT02848326 (MD-01), NCT03777059 (ADVANCE), NCT03700320 (study 302), NCT03939312 (study 309).

**Graphical Abstract:**

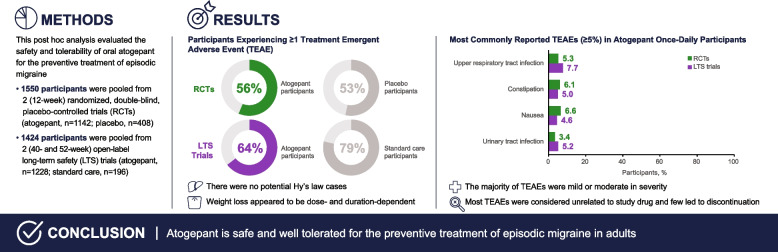

**Supplementary Information:**

The online version contains supplementary material available at 10.1186/s10194-024-01736-z.

## Introduction

Migraine, a leading cause of disability worldwide, is a highly prevalent neurologic disease defined by recurrent attacks of headache pain and associated symptoms (eg, photophobia, nausea, allodynia) [1]. According to the American Headache Society Consensus Statement, preventive treatment should be considered for individuals with migraine who experience ≥ 3 monthly headache days (MHDs) with severe disability, ≥ 4 MHDs with some disability, or ≥ 6 MHDs regardless of the level of disability [2]. Conventional non-specific preventive migraine treatments often demonstrate low rates of treatment persistence, primarily due to inadequate efficacy or poor tolerability [3]. Effective, well-tolerated, migraine-specific preventive treatments are needed to reduce migraine symptoms, improve function, and enhance quality of life [3, 4].

Atogepant, a migraine-specific oral CGRP receptor antagonist administered once daily, is indicated for the preventive treatment of migraine in adults [5]. Multiple clinical trials have confirmed its efficacy, safety, and tolerability [6–8]. In the phase 2b/3 (MD-01) and phase 3 (ADVANCE) trials, all studied doses of atogepant significantly reduced mean monthly migraine days across 12 weeks of treatment compared with placebo; the proportions of atogepant participants with ≥ 50% reduction in monthly migraine days across 12 weeks of treatment ranged from 52 to 62% [6, 7]. Moreover, in an open-label trial, 84% of atogepant participants achieved ≥ 50% reduction in monthly migraine days across 52 weeks of treatment [8]. This post hoc analysis using pooled data across four phase 3 clinical trials evaluated the overall safety and tolerability profile of atogepant for the preventive treatment of episodic migraine (EM) in adults, including the occurrence of adverse events (AEs) of interest. Based on the potential hepatotoxicity of first-generation gepants, treatment-emergent elevations in liver enzymes were evaluated as prespecified AEs of interest throughout the phase 3 clinical program of atogepant, a second-generation gepant [9, 10]. Given the potential effect on inhibition of vasodilation through the anti-CGRP mechanism of action, cardiac disorders, including hypertension, were also deemed AEs of interest [11, 12]. Identifying treatments that do not result in weight gain is important, as obesity is a common comorbidity and risk factor for disease progression in people with migraine [13]. Therefore, a change in body weight of ≥ 7% from baseline was determined as potentially clinically significant during the clinical trials and also an AE of interest.

## Methods

### Study design

This original research is a pooled analysis that assessed the safety and tolerability of atogepant in adults with EM using data from 2 randomized, double-blind, placebo-controlled trials (RCTs; phase 2b/3 [MD-01], NCT02848326; phase 3 [ADVANCE], NCT03777059) and 2 open-label long-term safety (LTS) trials (52 weeks, NCT03700320; 40 weeks, NCT03939312) (Table 1). The phase 2b/3 trial evaluated atogepant 10, 30, and 60 mg once daily and 30 and 60 mg twice daily versus placebo [6]. The ADVANCE trial evaluated the efficacy and safety of once-daily atogepant 10, 30, and 60 mg versus placebo [7]. Participants who completed one of these trials were eligible to participate in the LTS trials (phase 2b/3 and ADVANCE trial participants were eligible to enroll in the 52-week and 40-week LTS trials, respectively). Both LTS trials assessed the safety and tolerability of once-daily atogepant 60 mg [8, 14]. In the 52-week LTS trial, a standard care (SC) arm was included to contextualize long-term safety data [8]. Participants in the SC group could have been prescribed an alternative medication, not prescribed any migraine preventive medication, or discontinued migraine preventive medication as often as needed throughout the study. These results focused on the US-approved atogepant doses for EM of 10, 30, and 60 mg once daily, as twice-daily dosing is not approved [5].

**Table 1:** Atogepant clinical trials conducted in participants with episodic migraine

Study (ClinicalTrials.gov Identifier)	Phase	No. Treated	Study Design	Study Drug/Dose	Dosing Frequency	Study Visits
MD-01 (NCT02848326)	2b/3	825	Randomized, double-blind, placebo-controlled	Atogepant 10, 30, or 60 mg or placebo	All doses, once daily; 30 and 60 mg, twice daily	Weeks 2, 4, 6, 8, 12; follow-up week 16
ADVANCE (NCT03777059)	3	902	Randomized, double-blind, placebo-controlled	Atogepant 10, 30, or 60 mg or placebo	Once daily	Weeks 2, 4, 6, 8, 12; follow-up week 16
Study 302 (NCT03700320)	3	739	Randomized, open label	Atogepant 60 mg or standard care	Once daily	Weeks 4, 8, 12, 16, 20, 24, 28, 32, 36, 40, 44, 48, 52; follow-up week 56
Study 309 (NCT03939312)	3	655	Open label	Atogepant 60 mg	Once daily	Weeks 4, 8, 12, 16, 20, 24, 28, 32, 36, 40; follow-up week 44

All trials were performed in compliance with the International Conference on Harmonisation E6 Guideline for Good Clinical Practice and the principles of the Declaration of Helsinki. Each investigator obtained approval from an independent ethics committee or a local institutional review board. Before administration of any study procedures, participants provided written informed consent.

### Participants

Inclusion and exclusion criteria for each study were published previously [6–8, 14]. Eligible participants were adults with 4–14 migraine days per month in the 3 months before visit 1 and 4–14 migraine days in the 28-day baseline period. Participants were diagnosed with migraine with or without aura according to the International Classification of Headache Disorders, 3rd edition [15], > 1 year before screening, with diagnosis made before the age of 50 years.

### Safety parameters

Safety assessments were reported by participants throughout the trials and included evaluation of treatment-emergent AEs (TEAEs) for seriousness and relationship to study medication (as determined by the investigator), TEAEs leading to discontinuation, lab monitoring, electrocardiograms, and the Columbia Suicide Severity Rating Scale questionnaire. The intensity of each TEAE was assessed as mild (does not generally interfere with usual activities of daily living), moderate (interferes with usual activities of daily living), or severe (interrupts usual activities of daily living). Notably, TEAEs were not treatment related. Serious AEs (SAEs) were defined based on investigator judgement as those that were life-threatening or resulted in hospitalization, disability, or death, or events that may require medical or surgical intervention to prevent hospitalization, disability, or death. The following events were also highlighted due to clinical interest: alanine aminotransferase (ALT) or aspartate aminotransferase (AST) concentration ≥ 3 times the upper limit of normal (3 × ULN) and potential Hy’s law cases (defined as concurrent ALT or AST elevation ≥ 3 × ULN, bilirubin elevation ≥ 2 × ULN, and alkaline phosphatase value < 2 × ULN, in any of the clinical trials), weight changes, and cardiac disorders (including hypertension). Potentially clinically significant weight loss was defined as ≥ 7% reduction of body weight at any time postbaseline.

### Statistical analyses

Safety assessments were performed on the pooled safety population, which included participants who received ≥ 1 dose of study medication. AE data were collected through the last visit and are reported as number (percentage) of the pooled safety population (pooled RCTs and pooled LTS trials). AEs were classified by investigators by severity, potential relationship to study medication, start and stop dates, and seriousness. Demographics and baseline characteristics were summarized for the safety population. Descriptive statistics (n, mean, standard deviation [SD], median, minimum, and maximum) were presented for continuous variables. Frequency distributions (numbers and percentages) were reported for categorical variables.

## Results

### Participants

A total of 1550 participants were analyzed across the 2 pooled RCTs (Supplemental Fig. 1A; Table 1) and 1424 participants in the pooled LTS trials (Supplemental Fig. 1B; Table 1). Of the LTS trial participants, 792 were previously enrolled in an RCT (MD-01: 107; ADVANCE: 685). Demographic and baseline characteristics are summarized in Table 2.

**Table 2:** Demographic and baseline characteristics

	**Randomized, Placebo-Controlled Trials**				**Long-term Safety Trials**	
	**Atogepant**			**Placebo**	**Atogepant 60 mg once daily**	**Standard Care**
	**10 mg once daily**	**30 mg once daily**	**60 mg once daily**			
N	314	411	417	408	1228	196
Age, mean (SD), years	40.8 (12.18)	41.6 (12.58)	41.6 (12.14)	40.4 (12.29)	42.2 (12.18)	41.1 (12.09)
BMI, mean (SD), kg/m^2^	30.22 (7.49)	30.65 (7.42)	29.96 (7.54)	30.64 (8.22)	30.6 (7.65)	30.6 (8.03)
Female, n (%)	282 (89.8)	370 (90.0)	355 (85.1)	352 (86.3)	1083 (88.2)	172 (87.8)
Race, n (%)						
White	250 (79.6)	330 (80.3)	325 (77.9)	331 (81.1)	994 (80.9)	145 (74.0)
Black/African American	54 (17.2)	67 (16.3)	72 (17.3)	69 (16.9)	186 (15.1)	38 (19.4)
Asian	3 (1.0)	3 (0.7)	10 (2.4)	3 (0.7)	21 (1.7)	5 (2.6)
American Indian/Alaska Native	1 (0.3)	2 (0.5)	3 (0.7)	3 (0.7)	6 (0.5)	0 (0)
Native Hawaiian/Pacific Islander	0 (0)	2 (0.5)	0 (0)	0 (0)	2 (0.2)	2 (1.0)
Multiple races	6 (1.9)	7 (1.7)	6 (1.4)	2 (0.5)	18 (1.5)	5 (2.6)
Missing	0 (0)	0 (0)	1 (0.2)	0 (0)	1 (0.1)	1 (0.5)

*BMI* body mass index

### Treatment-emergent adverse events

TEAEs for the pooled safety population are summarized in Table 3. In the RCTs, 643/1142 (56.3%) atogepant participants and 218/408 (53.4%) placebo participants experienced ≥ 1 TEAEs. In the LTS trials, 792/1228 (64.5%) atogepant participants and 154/196 (78.6%) SC participants experienced ≥ 1 TEAEs.

**Table 3:** Summary of treatment-emergent adverse events for the pooled safety population

	**Randomized, Placebo-Controlled Trials**				**Long-term Safety Trials**	
	**Atogepant**				**Atogepant** **60 mg once daily** **(*n* = 1228)**	**Standard Care** **(*n* = 196)**
	**10 mg once daily** **(*n* = 314)**	**30 mg once daily** **(*n* = 411)**	**60 mg once daily (*n* = 417)**	**Placebo** **(*n* = 408)**		
Any TEAE, n (%)	178 (56.7)	234 (56.9)	231 (55.4)	218 (53.4)	792 (64.5)	154 (78.6)
Any study drug–related TEAE, n (%)	68 (21.7)	73 (17.8)	87 (20.9)	50 (12.3)	158 (12.9)	71 (36.2)
Any serious TEAE, n (%)	3 (1.0)	2 (0.5)	2 (0.5)	4 (1.0)	47 (3.8)^a^	7 (3.6)
Any TEAE leading to discontinuation, n (%)	13 (4.1)	14 (3.4)	12 (2.9)	11 (2.7)	53 (4.3)	5 (2.6)

The participants in the standard care arm could have been non-naive to their preventive treatment, could have selected their treatment based on their prior experience, and could switch preventive as needed; therefore, direct comparisons of adverse event rates between standard care and atogepant treatment arms cannot be made

*TEAE* treatment-emergent adverse event

^a^None due to atogepant

The most commonly reported TEAEs (≥ 5%) with once-daily atogepant were upper respiratory tract infection (5.3% in RCTs, 7.7% in LTS trials), constipation (6.1% in RCTs, 5.0% in LTS trials), nausea (6.6% in RCTs, 4.6% in LTS trials), and urinary tract infection (3.4% in RCTs, 5.2% in LTS trials). The majority of the TEAEs were mild or moderate in severity (Table 4).

**Table 4:** Treatment-emergent adverse events

	**Randomized, Placebo-Controlled Trials**				**Long-term Safety Trials**	
	**Atogepant**			**Placebo** **(*n* = 408)**	**Atogepant** **60 mg once daily** **(*n* = 1228)**	**Standard Care** **(*n* = 196)**
	**10 mg once daily** **(*n* = 314)**	**30 mg once daily** **(*n* = 411)**	**60 mg once daily** **(*n* = 417)**			
AEs (≥ 5% of atogepant-treated participants), n (%)						
Upper respiratory tract infection	15 (4.8)	27 (6.6)	19 (4.6)	25 (6.1)	94 (7.7)	24 (12.2)
*Mild*	7 (2.2)	14 (3.4)	11 (2.6)	12 (2.9)	44 (3.6)	16 (8.2)
*Moderate*	8 (2.5)	13 (3.2)	8 (1.9)	13 (3.2)	50 (4.1)	8 (4.1)
*Severe*	0	0	0	0	0	0
Constipation	19 (6.1)	26 (6.3)	25 (6.0)	5 (1.2)	62 (5.0)	6 (3.1)
*Mild*	16 (5.1)	19 (4.6)	17 (4.1)	3 (0.7)	41 (3.3)	4 (2.0)
*Moderate*	2 (0.6)	7 (1.7)	8 (1.9)	2 (0.5)	20 (1.6)	2 (1.0)
*Severe*	1 (0.3)	0	0	0	1 (0.1)	0
Nasopharyngitis	7 (2.2)	19 (4.6)	22 (5.3)	12 (2.9)	57 (4.6)	10 (5.1)
*Mild*	7 (2.2)	15 (3.6)	20 (4.8)	9 (2.2)	36 (2.9)	7 (3.6)
*Moderate*	0	4 (1.0)	2 (0.5)	3 (0.7)	21 (1.7)	3 (1.5)
*Severe*	0	0	0	0	0	0
Nausea	16 (5.1)	23 (5.6)	36 (8.6)	13 (3.2)	57 (4.6)	12 (6.1)
*Mild*	14 (4.5)	16 (3.9)	24 (5.8)	10 (2.5)	44 (3.6)	7 (3.6)
*Moderate*	2 (0.6)	6 (1.5)	11 (2.6)	3 (0.7)	13 (1.1)	5 (2.6)
*Severe*	0	1 (0.2)	1 (0.2)	0	0	0
Urinary tract infection	5 (1.6)	20 (4.9)	14 (3.4)	12 (2.9)	64 (5.2)	9 (4.6)
*Mild*	3 (1.0)	11 (2.7)	7 (1.7)	4 (1.0)	32 (2.6)	4 (2.0)
*Moderate*	2 (0.6)	9 (2.2)	7 (1.7)	8 (2.0)	32 (2.6)	4 (2.0)
*Severe*	0	0	0	0	0	1 (0.5)
AEs of interest, n (%)						
Weight decrease	1 (0.3)	0	4 (1.0)	3 (0.7)	32 (2.6)	3 (1.5)
*Mild*	1 (0.3)	0	2 (0.5)	1 (0.2)	26 (2.1)	2 (1.0)
*Moderate*	0	0	2 (0.5)	2 (0.5)	6 (0.5)	1 (0.5)
*Severe*	0	0	0	0	0	0
Weight increase	2 (0.6)	0	2 (0.5)	5 (1.2)	15 (1.2)	11 (5.6)
*Mild*	1 (0.3)	0	2 (0.5)	4 (1.0)	11 (0.9)	9 (4.6)
*Moderate*	1 (0.3)	0	0	1 (0.2)	4 (0.3)	2 (1.0)
*Severe*	0	0	0	0	0	0
ALT increase	5 (1.6)	4 (1.0)	6 (1.4)	9 (2.2)	16 (1.3)	4 (2.0)
*Mild*	1 (0.3)	1 (0.2)	4 (1.0)	5 (1.2)	8 (0.7)	2 (1.0)
*Moderate*	4 (1.3)	2 (0.5)	0	4 (1.0)	6 (0.5)	1 (0.5)
*Severe*	0	1 (0.2)	2 (0.5)	0	2 (0.2)	1 (0.5)
AST increase	4 (1.3)	4 (1.0)	4 (1.0)	8 (2.0)	20 (1.6)	5 (2.6)
*Mild*	2 (0.6)	2 (0.5)	1 (0.2)	3 (0.7)	10 (0.8)	1 (0.5)
*Moderate*	2 (0.6)	2 (0.5)	2 (0.5)	5 (1.2)	8 (0.7)	4 (2.0)
*Severe*	0	0	1 (0.2)	0	2 (0.2)	0
Hypertension	1 (0.3)	4 (1.0)	0	0	23 (1.9)	2 (1.0)
*Mild*	1 (0.3)	0	0	0	10 (0.8)	2 (1.0)
*Moderate*	0	4 (1.0)	0	0	13 (1.1)	0
*Severe*	0	0	0	0	0	0
Other cardiac disorder^a^	4 (1.3)	9 (2.2)	3 (0.7)	4 (1.0)	16 (1.3)	5 (2.6)
*Mild*	3 (1.0)	9 (2.2)	2 (0.5)	2 (0.5)	9 (0.7)	5 (2.6)
*Moderate*	1 (0.3)	0	1 (0.2)	2 (0.5)	7 (0.6)	0
*Severe*	0	0	0	0	0	0

Participants in the standard care arm were non-naive to their preventive treatment, selected their treatment based on their prior experience, and could switch preventive as needed; therefore, direct comparisons of AE rates between standard care and atogepant treatment arms cannot be made

*AE* Adverse event, *ALT* Alanine aminotransferase, *AST* Aspartate aminotransferase, *SC* Standard care

^a^Includes atrioventricular block first degree, atrioventricular block second degree, left bundle branch block, left atrial enlargement, palpitations, postural orthostatic tachycardia syndrome, and supraventricular extrasystoles

### Treatment-related TEAEs and serious TEAEs

Most TEAEs were considered not related to the study drug by the investigator in the RCTs and LTS trials. In the RCTs, 228/1142 (20.0%) atogepant participants and 50/408 (12.3%) placebo participants experienced ≥ 1 treatment-related TEAEs (TR-TEAEs). In the LTS trials, 158/1228 (12.9%) atogepant participants experienced ≥ 1 TR-TEAEs. The most commonly reported TR-TEAEs (> 2%) in atogepant participants were constipation (RCTs: 4.7% [54/1142]; LTS trials: 3.0% [37/1228]), nausea (RCTs: 4.0% [46/1142]; LTS trials: 2.0% [24/1228]), and fatigue (RCTs: 1.7% [19/1142]; LTS trials: 1.1% [14/1228]) (Table 5).

**Table 5:** Most common treatment-related treatment-emergent adverse events

Treatment-Related Adverse Event (> 2% of Atogepant-Treated Participants)	Randomized, Placebo-Controlled Trials				Long-term Safety Trials	
	**Atogepant**			**Placebo** **(*n* = 408)**	**Atogepant** **60 mg once daily** **(*n* = 1228)**	**Standard Care** **(*n* = 196)**
	**10 mg once daily** **(*n* = 314)**	**30 mg once daily** **(*n* = 411)**	**60 mg once daily** **(*n* = 417)**			
Constipation	14 (4.5)	20 (4.9)	20 (4.8)	3 (0.7)	37 (3.0)	3 (1.5)
Nausea	10 (3.2)	15 (3.6)	21 (5.0)	6 (1.5)	24 (2.0)	9 (4.6)
Fatigue	4 (1.3)	6 (1.5)	9 (2.2)	5 (1.2)	14 (1.1)	9 (4.6)
Somnolence	8 (2.5)	5 (1.2)	9 (2.2)	4 (1.0)	7 (0.6)	5 (2.6)

Serious TEAEs in the RCTs were reported in 7 (0.6%) atogepant participants compared with 4 (1.0%) in the pooled placebo group. In the LTS trials, 47 (3.8%) atogepant participants and 7 (3.6%) SC participants reported any serious TEAE (Table 3). With the exception of 1 case of optic neuritis, all serious TEAEs were considered by the investigator to be unrelated to study treatment. The case of optic neuritis occurred in a 23-year-old woman who received atogepant 10 mg once daily; magnetic resonance imaging of the brain and orbits, and visual-evoked potential results were normal, and the optic neuritis resolved without any sequela. Serious TEAEs are summarized in Table 6. In the LTS trials, 2 deaths were reported, 1 due to homicide and 1 due to beta-hemolytic streptococcal infection (toxic shock syndrome), both of which were deemed unrelated to atogepant per the investigator.

**Table 6:** Serious treatment-emergent adverse events

	**Randomized, Placebo-Controlled Trials**				**Long-term Safety Trials**	
	**Atogepant**			**Placebo** **(*n* = 408)**	**Atogepant** **60 mg once daily** **(*n* = 1228)**	**Standard Care** **(*n* = 196)**
	**10 mg once daily** **(*n* = 314)**	**30 mg once daily** **(*n* = 411)**	**60 mg once daily** **(*n* = 417)**			
Basal cell carcinoma	0	0	0	0	5 (0.4)	0
Abortion, induced^a^	0	0	1 (0.3)	0	2 (0.2)	0
COVID-19	0	0	0	0	2 (0.2)	0
Overdose	0	0	1 (0.2)	0	2 (0.2)	0
Road traffic accident	0	0	0	0	2 (0.2)	0
Noncardiac chest pain	0	0	0	0	1 (0.1)	2 (1.0)
Asthma	1 (0.3)	0	0	0	0	0
Brain injury	0	0	0	1 (0.2)	0	0
Cholecystitis	1 (0.3)	0	0	0	1 (0.1)	0
Gastric ulcer hemorrhage	0	0	0	1 (0.2)	0	0
Hodgkin disease	0	0	0	1 (0.2)	0	0
Major depression	0	0	1 (0.2)	0	0	0
Migraine	0	1 (0.2)	0	0	1 (0.1)	0
Negative-pressure pulmonary edema	0	0	0	1 (0.2)	0	0
Optic neuritis	1 (0.3)	0	0	0	0	0
Ureteritis	0	1 (0.2)	0	0	0	0
Ureterolithiasis	0	0	0	1 (0.2)	1 (0.1)	0

^a^Event specific to women. Percentages are based on number of female participants

### Treatment discontinuations

In the pooled RCTs, the proportions of participants with TEAEs leading to discontinuation were 3.5% (40/1142) with atogepant and 2.7% (11/408) with placebo. In the pooled LTS trials, 4.3% (53/1228) of atogepant participants and 2.6% (5/196) of SC participants discontinued due to TEAEs (Table 3). No dose–response relationship was observed for TEAEs leading to discontinuation. The AEs that most led to discontinuation in RCTs were constipation (0.5%), nausea (0.5%), and fatigue/somnolence (0.5%). The AEs that most led to discontinuation in the LTS trials were nausea (0.5%) and dizziness (0.3%).

### AEs of interest

AEs of interest included AEs commonly reported with atogepant and ones that are of interest to health care professionals (Table 4).

#### Constipation

Constipation was reported in 6.1% (70/1142) of atogepant participants in the RCTs and in 5.0% (62/1228) of atogepant participants in the LTS trials. Most cases of constipation were mild or moderate in severity and there was no clear dose dependency. There were no SAEs of constipation, including no constipation-related complications (eg, hospitalization, ileus). Few TEAEs of constipation resulted in discontinuation (RCTs: 0.5% [6/1142], LTS trials: 0.2% [2/1228]). Most cases (RCTs: 71.4% [50/70], LTS trials: 45.2% [28/62]) occurred within the first 2 weeks after treatment initiation, and the majority (RCTs: 64.3% [45/70], LTS trials: 87.1% [54/62]) resolved by the end of the study, either spontaneously or with over-the-counter treatment.

#### Nausea

Nausea was reported at rates of 6.6% (75/1142) in the RCTs and 4.6% (57/1228) in the LTS trials. The majority of cases were mild in severity. No SAEs of nausea occurred. Few instances of nausea led to discontinuation (RCTs: 0.4% [5/1142], LTS trials: 0.5% [6/1228]). Most cases (RCTs: 54.7% [41/75], LTS trials: 36.8% [21/57]) occurred within the first 2 weeks after treatment initiation, and the majority (RCTs: 94.7% [71/75], LTS trials: 96.5% [55/57]) of cases resolved by the end of the study.

#### Hepatic safety

Hepatic safety was evaluated thoroughly in all clinical trials through AE reporting and lab monitoring. An external clinical adjudication committee provided a blinded review and adjudication of all post-treatment elevations of ALT or AST ≥ 3 × ULN to assess their causal relationship to atogepant. In the RCTs, 1.3% (15/1142) and 1.1% (12/1142) of atogepant participants experienced a TEAE of increased ALT or AST, respectively (placebo: ALT, 2.2% [9/408]; AST, 2.0% [8/408]). In the LTS trials, 1.3% (16/1228) and 1.6% (20/1228) experienced a TEAE of increased ALT or AST, respectively (SC: ALT, 2.0% [4/196]; AST, 2.6% [5/196]) (Table 4).

There were no potential Hy’s law cases. In the RCTs, the rates of ALT or AST elevation ≥ 3 × ULN were similar between those who received atogepant (1.0% [11/1126]) and those who received placebo (1.8% [7/399]). In the LTS trials, ALT or AST elevation ≥ 3 × ULN was observed in 1.4% (17/1214) with atogepant and 3.2% (6/190) with SC. The adjudication committee determined most cases to be unlikely related to atogepant and a few to be probably or possibly (8 cases in RCTs and LTS trials) related to atogepant. Among the possible/probable cases, confounding factors were also identified in all but 1 case.

#### Weight changes

In the RCTs, the percentages of participants with a weight decrease of a clinically significant weight loss of ≥ 7% at any time point during the 12 weeks were 3.8% for atogepant 10 mg, 3.2% for atogepant 30 mg, and 4.9% for atogepant 60 mg, compared with 2.8% for placebo. At week 12, the percentage changes from baseline for atogepant 10 mg, 30 mg, and 60 mg were 0.12, − 0.51, and − 1.02. In the LTS trials, the percentages of clinically significant weight loss were 14.7% for those treated with SC and 24.1% for those treated with atogepant 60 mg once daily. At week 4 and week 40, the percentage changes from baseline for atogepant 60 mg were − 0.42 and − 2.38, respectively, in the 52-week LTS trial, and − 0.76 and − 2.09, respectively, in the 40-week LTS trial. Weight loss appeared to be dose- and duration-dependent.

The percentages of atogepant participants who reported a TEAE of decreased weight were 0.4% in the RCTs and 2.6% in the LTS trials (Table 4). Discontinuation due to weight loss as a TEAE was rare (0 in the RCTs and 2 participants in the LTS trials). An exploratory analysis revealed no clear relationship between weight change and nausea or vomiting, but a slightly higher incidence of decreased appetite was observed in weight-loss responders.

#### Cardiac Disorders and Hypertension

In the RCTs and LTS trials, cardiac disorders were uncommon TEAEs. Hypertension was reported by 0.4% (5/1142) of atogepant participants and 0% of placebo participants in the RCTs, and by 1.9% (23/1228) of atogepant participants and 1.0% (2/196) of SC participants in the LTS trials (Table 4). There were no serious cases of hypertension in any of the clinical trials. One participant in each of the pooled RCTs and pooled LTS trials discontinued due to hypertension. No cases in the RCTs were considered treatment related. One nonserious case of mild hypertension in the LTS trials was considered related to atogepant treatment by the investigator. Blood pressure was measured at each participant visit, and the data collected across RCTs and LTS trials did not reveal any clinically relevant changes in blood pressure. Other cardiac disorders (including atrioventricular block first degree, atrioventricular block second degree, left bundle branch block, left atrial enlargement, palpitations, postural orthostatic tachycardia syndrome, and supraventricular extrasystoles) were experienced by 1.4% (16/1142) of atogepant participants and 1.0% (4/408) of placebo participants in the RCTs and by 1.3% (16/1228) of atogepant participants and 2.6% (5/196) of SC participants in the LTS trials.

## Discussion

Preventive migraine therapies have been underutilized at least in part due to limited efficacy and poor tolerability of the oral nonspecific generic treatments [3]. Atogepant is a migraine-specific preventive treatment that has demonstrated rapid and continuous reductions in mean monthly migraine days among adults with episodic and chronic migraine [6, 7, 16, 17]. Analyses of pooled data from 4 phase 3 clinical studies [2, 6, 8, 14] indicate that once-daily atogepant may offer a more favorable safety and tolerability profile for the preventive treatment of EM in adults. Most TEAEs in the 4 clinical studies were mild to moderate in severity and considered unrelated to the study drug. The proportions of serious TEAEs were similar in the atogepant and placebo groups.

Among frequently reported TEAEs were constipation, nausea, and fatigue/somnolence, with most cases mild or moderate in severity and managed through standard clinical practice or resolving spontaneously. As atogepant can be administered with or without food [5], the incidence of nausea might be reduced by taking it with food.

In all four clinical trials, no potential Hy’s law cases were reported. A few cases of ALT/AST elevations ≥ 3 × ULN were temporally associated with atogepant, but all were nonserious, mild to moderate in severity, without concurrent bilirubin elevations, and resolved with or without atogepant discontinuation. No dose adjustments are required for mild or moderate hepatic impairment [5]. However, atogepant is not recommended in individuals with known severe hepatic impairment.

The administration of atogepant was associated with modest dose- and duration-dependent weight loss, without a significant clinical impact, as reflected by the low adverse event reporting rates and study drug discontinuation. The underlying mechanism for this observation remains unknown.

This analysis showed a low incidence of cardiovascular AEs associated with atogepant, which is consistent with the published literature for other anti-CGRP therapies [18, 19]. However, as CGRP is a potent vasodilator [20], concerns exist regarding the use of therapies that target the CGRP pathway and blood pressure regulation. In a prospective follow-up study of anti-CGRP mAbs, increased mean systolic and diastolic blood pressure was observed after 3 months of treatment and continued throughout 12 months [11]. Although our findings show that 5 participants treated with atogepant experienced a TEAE of hypertension in the RCTs, none of the cases were deemed to be treatment related. In a post hoc analysis of participants with cardiovascular risk factors, without significant cardiovascular diseases, a low incidence of overall cardiovascular TEAEs was observed, and none were serious [21]. These results suggest the safety of atogepant in those with cardiovascular risk factors. Additionally, AEs of atogepant tend to resolve rapidly upon its discontinuation due to its half-life of approximately 11 h and elimination within approximately 2 days [5].

Pooled analyses increase the power to detect potentially rare events that may have been missed in single studies, but they have limitations [22]. This pooled analysis included participants with 4–14 migraine days per month and hence excluded participants with chronic migraine, limiting generalizability. However, a separate clinical trial of atogepant for the preventive treatment of chronic migraine has demonstrated positive efficacy results and a similar safety and tolerability profile [16]. Moreover, participants demonstrating greater tolerability in the RCTs were more prone to enroll in the LTS trials. Furthermore, the LTS trials were open-label trials that may have had potential inherent biases. Lastly, we acknowledge that additional studies are warranted to further evaluate the safety of atogepant in a more diverse patient population.

## Conclusion

In summary, atogepant showed a favorable safety and tolerability profile in adults with EM. The most common TEAEs associated with atogepant were mild to moderate constipation, nausea, fatigue, and somnolence that did not lead to discontinuation. Taken together with existing data on the efficacy and safety of atogepant, this analysis further supports the safety and tolerability of atogepant for the preventive treatment of EM.

### Supplementary Information

**Additional file 1: Supplemental Figure 1. **Participant disposition in the randomized, placebo-controlled (A) and long-term safety (B) trials.

## Data Availability

AbbVie is committed to responsible data sharing regarding the clinical trials we sponsor. This includes access to anonymized, individual, and trial-level data (analysis data sets), as well as other information (eg, protocols, clinical study reports, or analysis plans), as long as the trials are not part of an ongoing or planned regulatory submission. This includes requests for clinical trial data for unlicensed products and indications. These clinical trial data can be requested by any qualified researchers who engage in rigorous, independent, scientific research, and will be provided following review and approval of a research proposal, Statistical Analysis Plan, and execution of a Data Sharing Agreement. Data requests can be submitted at any time after approval in the US and Europe and after acceptance of this manuscript for publication. The data will be accessible for 12 months, with possible extensions considered. For more information on the process or to submit a request, visit the following link: https://vivli.org/ourmember/abbvie/ then select “Home.”

## Abbreviations

AE: Adverse event
ALT: Alanine aminotransferase
AST: Aspartate aminotransferase
CGRP: Calcitonin gene–related peptide
EM: Episodic migraine
LTS: Long-term safety
RCT: Randomized, double-blind, placebo-controlled trial
SC: Standard care
TEAE: Treatment-emergent adverse event
TR-TEAE: Treatment-related treatment-emergent adverse event
ULN: Upper limit of normal
