# Supplementary material for: Safety and tolerability of atogepant for the preventive treatment of migraine: a post hoc analysis of pooled data from four clinical trials
Source: J Headache Pain. 2024 Mar 11;25(1):35. doi: 10.1186/s10194-024-01736-z (PMC10926658; doi:10.1186/s10194-024-01736-z)

**Supplemental Figure 1.** Participant disposition in the randomized, placebo-controlled (A) and long-term safety (B) trials


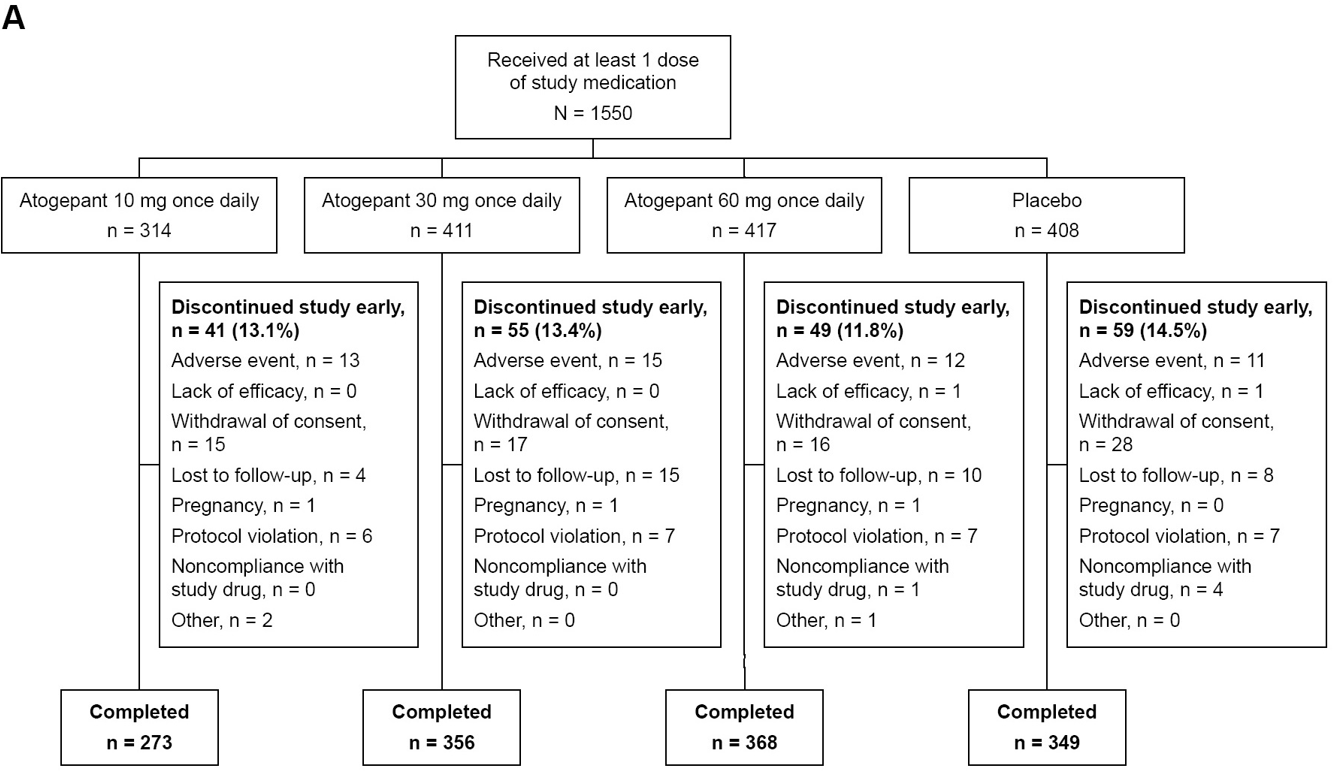


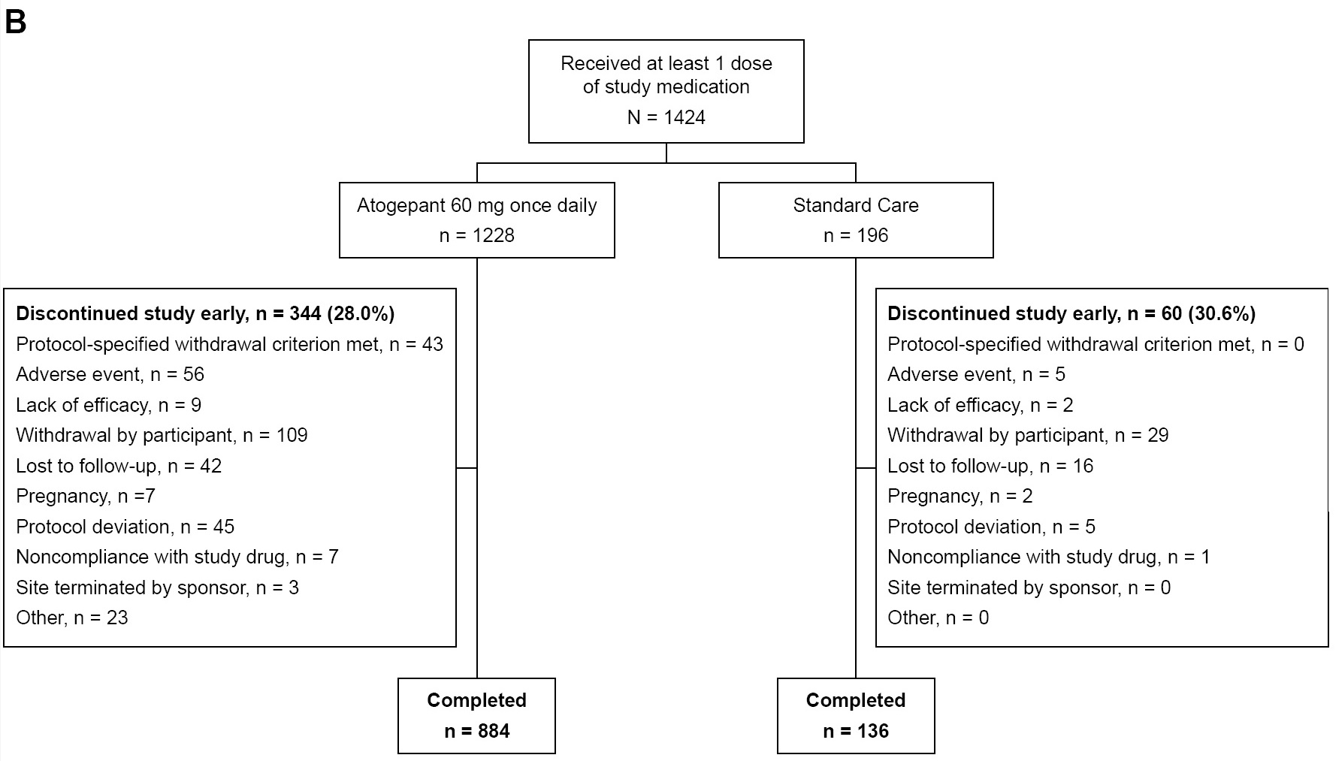

Supplement: Supplementary file 1 — Additional file 1: Supplemental Figure 1. Participant disposition in the randomized, placebo-controlled (A) and long-term safety (B) trials. [file 10194_2024_1736_MOESM1_ESM.docx]
